# Advances in Membrane Separation for Biomaterial Dewatering

**DOI:** 10.1021/acs.langmuir.3c03439

**Published:** 2024-02-22

**Authors:** Esli Diepenbroek, Sarthak Mehta, Zandrie Borneman, Mark A. Hempenius, E. Stefan Kooij, Kitty Nijmeijer, Sissi de Beer

**Affiliations:** † Department of Molecules & Materials, MESA+ Institute, University of Twente, 7500 AE Enschede, The Netherlands; ‡ Membrane Materials and Processes, Department of Chemical Engineering and Chemistry, Eindhoven University of Technology, 5600 MB Eindhoven, The Netherlands; § Physics of Interfaces and Nanomaterials, MESA+ Institute, University of Twente, 7500 AE Enschede, The Netherlands

## Abstract

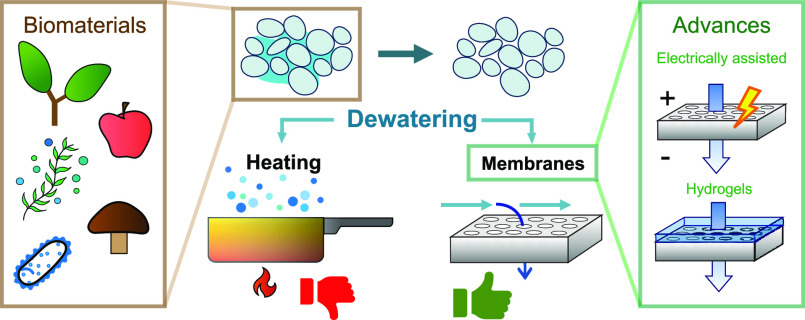

Biomaterials often contain large quantities of water (50–98%), and with the current transition to a more biobased economy, drying these materials will become increasingly important. Contrary to the standard, thermodynamically inefficient chemical and thermal drying methods, dewatering by membrane separation will provide a sustainable and efficient alternative. However, biomaterials can easily foul membrane surfaces, which is detrimental to the performance of current membrane separations. Improving the antifouling properties of such membranes is a key challenge. Other recent research has been dedicated to enhancing the permeate flux and selectivity. In this review, we present a comprehensive overview of the design requirements for and recent advances in dewatering of biomaterials using membranes. These recent developments offer a viable solution to the challenges of fouling and suboptimal performances. We focus on two emerging development strategies, which are the use of electric-field-assisted dewatering and surface functionalizations, in particular with hydrogels. Our overview concludes with a critical mention of the remaining challenges and possible research directions within these subfields.

## 1 Introduction

Within the current transition to a biobased economy, many challenges must be resolved.^1^ A critical challenge is that biobased materials often contain large quantities of water (50–98%).^2−4^ Biomaterials are materials derived from land-based and aquatic plants, animals, bacteria, and fungi.^5^ Their large moisture content makes transport and processing cost- and energy-intensive, making efficient dewatering an essential unit operation in biorefineries.^6,7^ Yet, traditional dewatering techniques based on thermal or chemical drying are thermodynamically inefficient and, currently, account for 15% of the energy consumed in industry.^2,3,8^ Moreover, these techniques can negatively affect the product quality.^9,10^ Dewatering through membrane separation will provide an energy-efficient alternative and has therefore gained a lot of attention in recent years.^11−15^

Membranes allow for selective water filtration by means of size exclusion, solution diffusion, and solute–membrane affinity,^16,17^ hereby separating the smaller water molecules from the larger biomaterial constituents. Membrane separations are used for many applications, ranging from water purification,^18−20^ gas separations,^21,22^ oil–water separations,^23−25^ and fuel cells^26−29^ to biomedical separations,^30−32^ and extensive literature can be found on the design and performance of these membranes. However, the utilization of membranes in the dewatering of biomaterials introduces new requirements and challenges that need to be solved.

**Figure 1:**
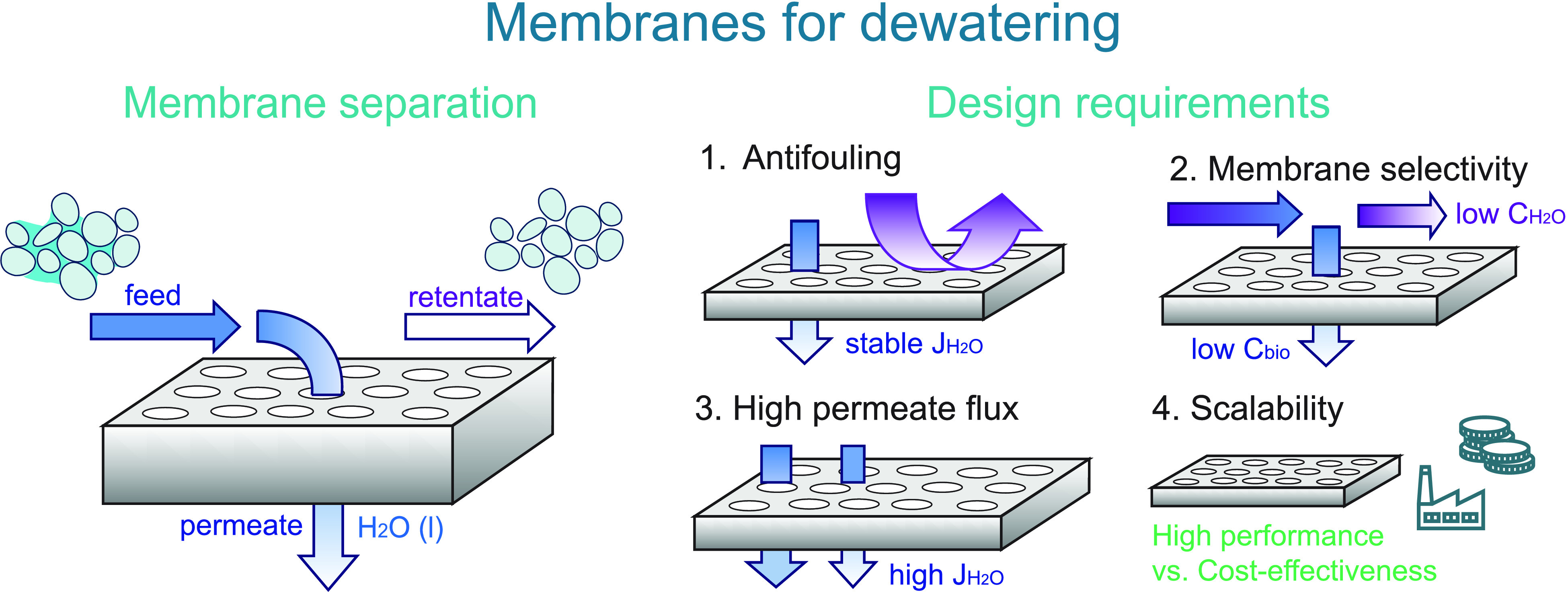
A conceptual overview of biomaterial dewatering through membrane separation (left) and the four design requirements for an efficient membrane separation (right). Requirements for membrane dewatering for biomaterials are listed as (1) antifouling properties, (2) membrane selectivity, (3) high permeate flux, and (4) scalability. The parameters *J*_H_2_O_, *C*_H_2_O_, and *C*_bio_ depict the water flux and concentrations of water and biomaterials, respectively.

To achieve a satisfactory performance for biomaterial dewatering, researchers have been developing suitable membrane separations for various classes of biomaterials. Extensive literature can be found on the topic of microalgae harvesting,^13,33,34^ protein concentration,^35−37^ and polysaccharide removal.^38^ All of these dewatering applications require a high water/biomaterial selectivity and permeate flux, combined with minimal fouling (Figure 1).^39,40^ However, maintaining a high permeate flux over time is a major challenge and depends on the antifouling properties of the membrane.^41−43^ Contrary to membrane separations in which the permeate is the product, biomaterial dewatering typically yields a retentate product. In such cases the quality of the retentate needs to be maintained, therewith raising the need for different antifouling strategies.^8,44−47^ Recent studies in this research field aim to improve current membrane materials by focusing on improving the dewatering performance and antifouling properties.^48−52^ In addition, membrane separations have been performed under applied vacuum^13,34,53^ and under the influence of vibrations^54,55^ and an electric field^56,57^ to improve the overall performance for biomaterial dewatering.

In this review, we will first discuss the major design requirements for the dewatering of biomaterials using membranes (section 2), followed by the recent developments that address the requirements and challenges mentioned above (section 3). These developments have focused on two strategies: (section 3.1) using an electrical driving force to tune the interaction of biomaterials with the membrane interface and enhance the selective permeation of water, and (section 3.2) surface functionalization, in particular with hydrogels, which are used to counteract fouling and increase selectivity and flux. Alongside an overview of the existing literature, we identify future challenges and knowledge gaps within these membrane material developments. Though reviews on membrane dewatering of biomaterials have been published, they focus either on specific feeds^2,3,44,58,59^ or primarily on fouling prevention.^48,60−63^ As far as we are aware, we are the first to provide an overview of the design requirements and developments of the complete field.

## 2 Requirements for Membrane Design

### 2.1 Performance Requirements

#### 2.1.1 Permeate Flux and Membrane Selectivity

The dewatering performance of a membrane is given by three figures of merit: (1) the permeate flux; (2) the selectivity, i.e., the ratio of permeation of the different components; and (3) the retention, i.e., the ratio of a component in the permeate divided by the concentration in the feed.^64^ These important parameters are dictated by the membrane geometry and morphology, such as the thickness, pore size, pore size distribution, and porosity. Simultaneously, material properties like the charge density and hydrophilicity also influence the dewatering performance. These parameters control not only the dewatering performance but also the fouling tendency, swelling, chemical and cleaning stability, and lifetime of the membranes.^65−67^ In the next sections, the contributions of the aforementioned parameters to the dewatering efficiency will be discussed in more detail.

#### 2.1.2 Membrane Fouling

**Figure 2:**
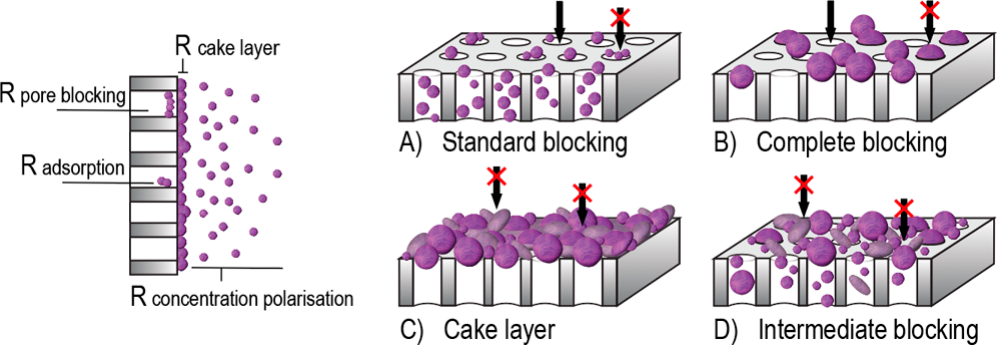
An overview of membrane fouling. Left: Resistances (*R*) to water flux due to membrane fouling. Right: Different mechanisms for membrane fouling: (A) standard blocking of membrane pores by small(er) biomaterial particles; (B) complete blocking of membrane pores by large biomaterial particles; (C) cake layer formation on the top surface layer; (D) intermediate blocking of biomaterials in membrane pores and on the top surface layer.

Membrane fouling is defined as the adsorption or deposition of particles and solutes on membrane surfaces^40,51^ and within pores.^3,68^ This leads to a reduction in permeate flux and negatively affects membrane selectivity as well.^69^ The decrease in flux during dewatering can be caused by either long-term irreversible fouling or reversible fouling, which is a directly occurring phenomenon. The resistances toward mass transport that may occur during a dewatering process are schematically represented in Figure 2.

While reversible fouling can be solved by back washing or forward flushing, irreversible fouling requires chemical cleaning. Concentration polarization is considered to be an example of reversible fouling.^70^ This type of fouling can be controlled by hydrodynamic processes, such as turbulent flow conditions, mixing, and reduction of boundary layer effects, which effectively increase the apparent retention and the transmembrane flux. On the otherhand, the prevention of irreversible fouling is preferred, since the chemical cleaning treatment introduces an additional, costly to dispose of, waste stream and also reduces the membrane lifetime. For this reason, the choice of membrane material is important in order to minimize unfavorable membrane–foulant interactions.^60,71−73^ Foulant–foulant interactions can also impact membrane fouling. Meng et al.^74^ provide a useful database of different polysaccharides and their fouling mechanisms. The study used Hermia’s model theory^75^ to classify fouling into complete blocking, intermediate blocking, standard blocking, and cake layer formation (see also: Figure 2). The study emphasized the development of agglomerates of polysaccharides in the feed mixture, due to foulant–foulant interactions in the feed.^76,77^ This suggests that thorough mixing of the feed is essential to decrease fouling and consequently achieve a high permeate flux.

Sadare et al.^78^ developed membranes with enhanced antifouling properties for the separation of succinate from a fermentation broth. Membranes were made of a PSF/PES polymer blend. The membranes were reported to be hydrophilic, which was enhanced further by a PVA surface coating. Scanning electron microscopy revealed an asymmetric pore size distribution along the membrane cross section. The dense top layer appeared to prevent pore plugging, positively impacting the permeate flux. Optimizing the membrane chemistry led to an increase in the flux recovery ratio (FRR), suggesting its influence on membrane–foulant interactions. It was found that the FFR was more connected to the pore size than to the hydrophilicity of the membranes. Similarly, Shi et al.^79^ coated a hydrophobic PVDF membrane with a hydrophilic rhamnolipid biosurfactant. Compared to pristine PVDF membranes, the rhamnolipid coated membranes showed a reduction in contact angle from 74 to 5.5° and smaller pore sizes. Subsequent filtration experiments with a BSA solution and a fermentation broth showed an improvement in the antifouling performance, indicating that hydrophilicity has a beneficial effect on the antifouling properties of membranes.

### 2.2 Membrane Chemistry

Membrane chemistry has a profound impact on the membrane performance for dewatering. Intermolecular interactions between the biomaterial and membrane surfaces are highly influenced by the membrane chemistry and ultimately impact the membrane performance and selective permeation of biomaterial components.^80−82^ Additionally, membrane chemistry not only determines interactions at the interface, it also influences the membrane stability and its resistance against cleaning agents and high pressures.^83,84^ Membranes are commonly synthesized from organic polymeric materials. Most frequently used are poly(ether sulfone) (PES),^85,86^ polyvinyldene fluoride (PVDF),^87,88^ regenerated cellulose (RC),^89,90^ regenerated cellulose acetate (RCA),^80^ poly(vinyl alcohol) (PVA),^91−95^ polyacrylonitrile (PAN),^13,96,97^ polyamide (PA),^39,81,82,98^ cellulose ester (CE),^99^ and polysulfone (PS).^80^ In the following subsections we briefly review their properties, the enabling chemistry, and their impact on the dewatering performance of the membrane.

#### 2.2.1 Hydrophilicity

**Figure 3:**
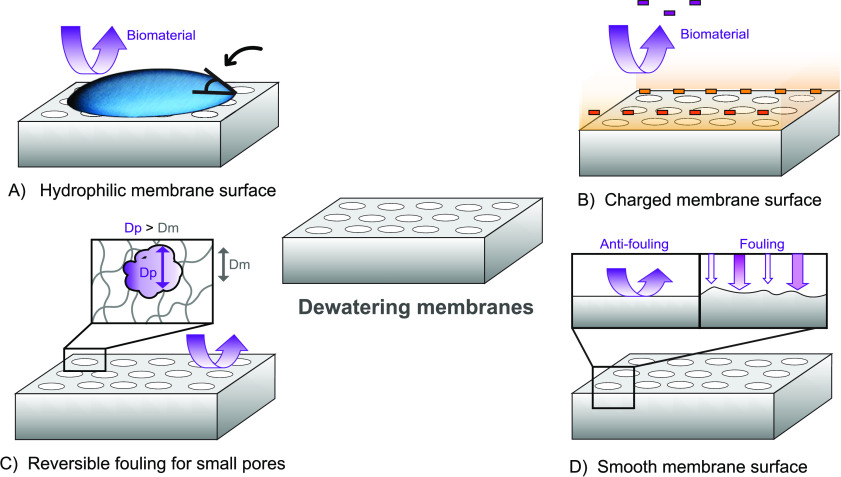
Desired membrane properties for enhanced dewatering performance: (A) hydrophilic membrane surface for improved antifouling; (B) charged membrane surfaces to repel similarly charged biomass particles; (C) pore size smaller than biomass particles to reduce irreversible fouling; (D) smooth membrane surface to reduce surface area and therewith foulant adsorption.

The smaller contact angles of hydrophilic materials indicate wettability, which produces a thin liquid layer on the membrane surface.^100^ As shown in Figure 3A, this liquid layer protects the membrane surface and pores from being fouled or clogged, preventing absorption of biomaterial components on the surface.^90,101^ Hydrophilicity also has an impact on permeate flux. Miao et al.^102^ present an optimized approach for hydrophilic modifications. A membrane performs best when it is adequately wetted by the feed solution at the desired operating pressure. This condition can be achieved by selecting the appropriate membrane chemistry or modifying a membrane’s chemical characteristics.^39,63,80,103^

A comparative study between PAN and PES membranes conducted by Rossi et al.^97^ reports a higher permeate flux for PAN-based membranes when used for microalgae dewatering. This was attributed to the more hydrophilic nature of PAN. Another study focused on blending biopolymers with PES and tested their hydrophilic contribution to the overall membrane performance.^104^ The resulting ultrafiltration (UF) membrane enabled separation of acidic media from saccharides. Hydrophilicity can also be introduced by surface modification, leading to enhanced antifouling abilities.^105,106^ The following subsection focuses on surface functionalization, which gives rise to multiple benefits for both chemical and morphological properties of the membrane.

#### 2.2.2 Surface Functionalization

Surface functionalization allows for the introduction of surface moieties different from those of the bulk membrane. This helps optimize membrane properties more independently of each other to improve the overall dewatering performance.^107^

Surface functionalization has been commonly studied to introduce hydrophilicity to the membrane surface. For instance, depositing inorganic particles such as TiO_2_^98,102^ and ZnO^108^ have been reported to increase the membrane’s affinity to water. Hydrophilic polymeric coatings and grafting on membrane surfaces are other techniques that improve the dewatering performance.^109^ Huang et al.^110^ treated pristine PVDF membranes with amine-functionalized silica groups. This treatment decreased the contact angle by a factor of 2. However, prolonged treatment during membrane functionalization induced hydrolysis, resulting in poor performance. Experimental studies also show that grafting can be done on the membrane fabrication reagent before preparing membranes.^111^ Optimizing the interactions between the solvent, polymer coating, and membrane material during the coating of membranes is a strategy to produce desirable surface properties.^112^ For example, Louie et al.^113^ coated a TFC-aromatic PA membrane with a 1 wt % PEBAX solution. After solvent evaporation, the PEBAX coating resulted in intrinsic bonding between PEBAX coating and subsequently a reduced pore size and lower permeate flux.

Apart from chemical attributes, surface functionalization can also improve morphological characteristics. Liu et al.^114^ modified a PA membrane by grafting PVA chains on the membrane surface. Grafting the valley and ridge surface structures with PVA led to a reduction in the surface roughness by 18.2%. This significantly enhanced the antifouling properties due to the synergistic effect of increased hydrophilicity and decreased surface roughness.^115^

#### 2.2.3 Modification of Surface Charges

Surface charge modification is a special case of surface functionalization. Functionalizing the membrane with specific chemical moieties allows us to control interactions between the membrane surface and charged biomass particles. Tuning Coulombic interactions between the foulants and membrane surface can significantly improve the membrane performance by preventing adsorption and blocking of pores.^82,116−118^ as shown in Figure 3B. Similar conclusions were drawn from a study by Akbari et al.,^81^ in which a PA membrane was coated with a cationic polysaccharide, resulting in a 50% increase in permeate flux when tested against a feed containing cationic surfactants. The extent of charged coating is strongly dependent on the membrane morphology.^119^ The study reports that larger pore size membranes allowed for better permeation of the polyelectrolyte, hereby producing a charged coating on both the pore wall and membrane surface. On the other hand, smaller pore sizes resulted in top layer formation only. The former was reported to significantly increase salt rejection due to improved Donnan exclusion inside the pores.

Generally, membranes with a specific charge can repel only similarly charged foulants while attracting oppositely charged ones. This is especially relevant for heterogeneous feeds and more extreme operating conditions, which give rise to significant fouling. Introducing an electrically neutral polymer on the inherently charged membranes can reduce the electrostatic interactions, making them versatile against a broad range of foulants. This leads to a decrease in irreversible fouling and higher FRR values.^120^ The amphiphilic nature of zwitterionic coatings can provide the intended effect for a broad range of operation conditions.^96,121−127^

### 2.3 Morphological Requirements

Dewatering biomaterials with the use of membranes relies on the physical separation of two phases. This is enabled by the morphology of the membrane, consisting of a surface layer and a supporting layer. The surface layer is characterized by pore size, porosity, tortuosity, and surface roughness, and it dictates the selectivity and permeate flux of the membrane.^64,65,128^ The surface layer is thin and is supported by a supporting layer. An ideal supporting layer should provide sufficient mechanical strength to the membrane with minimal contribution to the mass flow resistance. This is done by designing the pore size of the supporting layer much larger than those in the surface layer and having good interpore connectivity.^78,129,130^ Given the function of each layer, it is essential to optimize both the surface and supporting layers in order to optimize the membrane dewatering performance.

#### 2.3.1 Membrane Pore Size and Porosity

Porous membranes used for biomaterial dewatering commonly rely on size exclusion. This is determined by the membrane pore size and shape. Porous membranes are classified on the basis of their pore size into nanofiltration (NF), UF, and microfiltration (MF).^64^ Studies suggest that NF or dense UF membranes are commonly used for concentrating biomass.^3,68,87,130−133^ The pore sizes of these membranes are usually smaller than the biomass particles, herewith preventing complete or standard blocking, as shown in Figures 2A,B and 3C.^134^ This leaves cake layer formation as the primary fouling mechanism.^74^ The filtration resistance of this cake layer can be controlled by hydrodynamic conditions^129^ as well as back and forward flushing.^40,51,135,136^

Membrane volume porosity is defined as the volume fraction of the empty voids present in the total volume of the membrane.^137^ The empty voids (pores) in the membrane enable water permeation, making it an essential membrane property to control. A higher membrane volume porosity typically leads to bigger surface pores, which reduces the membrane selectivity and herewith the filtration performance.^65^ Novel membrane formation techniques have been developed to decouple pore size and porosity.^138^

While membranes with a high surface porosity, a narrow pore size distribution, and an interconnected pore structure are preferred for their high permeation and selectivity, Hwang et al.^129^ offers a different perspective. They conducted fouling experiments comparing spongelike structured pores to uniform straight-through circular pores. It was revealed that, compared to spongelike membranes, straight-through pores had negligible pore plugging. This suggests that the high porosity of a spongelike structure leads to open top layers, which are more prone to pore plugging. He et al.^65^ suggests a synthesis method to decouple surface porosity and pore sizes. By introducing Pluronic F127 to the polymeric dope solution and tannin (TA) to the bath, the study reports a thorough control over pore nucleation density and growth rate. Optimizing the concentrations of the mentioned additives led to a membrane with a smaller pore size distribution and an increased surface porosity. The same was reflected in performance testing as the flux increased by a factor of 2.

#### 2.3.2 Surface Roughness

The impact of surface roughness on the filtration performance is relatively unexplored. This is because the effect of surface roughness on the membrane performance is not an isolated phenomenon. It is accompanied by chemical and electrochemical interactions between the foulant and the membrane surface.^139^ A change in surface roughness often relates to a change in hydrophilicity/hydrophobicity, surface charge, pore size, and porosity.^114^ While Jiang et al.^140^ reports an insignificant contribution of surface roughness to the overall membrane performance, other studies show that the surface roughness does have a significant effect on fouling and the overall performance. Tong et al.^141^ reports that initial adhesion of biomass particles on membrane surfaces is mainly mediated by electrostatic forces, van der Waals forces, and acid–base interactions. A high surface roughness facilitates foulant attraction and adhesion.

Hoek et al.^142^ suggest that surface roughness at the nanometer scale is significant for intermolecular interactions between biomass particles and membrane surface. The widely known Derjaguin–Landau–Verwey–Overbeek (DLVO) model suggests that, in close proximity to a rough surface, a foulant particle encounters a high number of protruding surfaces, which lowers the repulsive interaction energy. In a typical hill and valley structure of an interfacial polymerization membrane, the low repulsive energy causes such foulant deposition (Figure 3D). He et al.^143^ stated that membranes with fewer crevices are less sensitive to biofouling. Surface modification is an excellent way to reduce surface roughness and its tendency to foul.^114^ While the above examples provide a generic and direct correlation between surface roughness and fouling properties, Horseman et al.^144^ use the DLVO model in combination with membrane surface chemistry. The study suggests that, while DLVO correlates attraction of a specific component to the membrane surface, it is the chemistry selectivity that defines which components are attracted to the membrane. In the case of hydrophilic membranes, surface roughness synergistically contributes in the reduction of fouling, by attracting a tight water layer on the surface.^100^ On the other hand, hydrophobic surfaces with high surface roughness have an increased tendency to foul. This conclusion relates to our initial statement: while surface roughness contributes to membrane performance, its effect cannot be studied in isolation.

### 2.4 Toward Applications

As mentioned in the previous sections, a high performance for dewatering relates to a high permeate flux, high biomaterial retention, and low fouling (section 2.1). This is enabled by a membrane design in which the chemistry is optimized (section 2.2) as well as the morphology (section 2.3). Although there are several studies identifying suitable membranes for biomaterial dewatering, large scale implementation remains a significant challenge.^145,146^

Reduction of permeate flux over prolonged operation makes the process inefficient and financially infeasible.^34^ In order to maintain the desired permeate flux, membranes are periodically flushed,^135,136^ which often leads to redilution of the feed.^33^ In the case of more adhesive fouling, harsh cleaning agents are used that can destroy the membrane.^69,147^ Working with foulant-rich media such as biomaterial streams also reduces the membrane lifespan.^148^ All these cases suggest that, while membrane dewatering is a sustainable solution, it still faces several challenges for large scale implementation.

Literature suggests that the highest contribution to membrane fouling is the transmembrane pressure applied as a driving force.^19,149,150^ One of the alternatives to pressure driven systems is forward osmotic (FO) dewatering.^151^ A bibliometric analysis on the concentration of liquid foods^152^ reports that FO is one of the most studied processes to concentrate such slurries. However, FO systems have their own challenge such as internal concentration polarization (ICP).^153−155^ Moreover, most studies on FO dewatering suggest that this technology is better suited for the dewatering of diluted biomass (2–15 °Brix), allowing for a higher dewatering rate and low fouling sensitivity.^85,151,156,157^ Most studies on FO dewatering lie in the range of 4–7 on the technology readiness level (TRL), which means that they are successful on the laboratory scale and start to enter pilot scale testing.^152^

To summarize, dewatering using membranes still faces a lot of technical challenges. From excessive fouling leading to poor dewatering performance, to the inability to dewater semidiluted or concentrated biomaterial streams, membrane technology needs significant improvements for wide-scale implementation in the bioprocessing industry. In section 3 we will discuss recent developments in membrane technology that attempt to tackle these problems and have the potential to improve membrane-assisted dewatering performances.

## 3 Advances in Membrane Dewatering

### 3.1 Electrically Assisted Membrane Dewatering

Biomaterials have the ability to produce surface charges (measured as zeta potential) when in direct contact with an aqueous medium. As a result, biomass particles are surrounded by an electric double layer (EDL) at the biomass–water interface.^158^ The developed EDL consists of opposite charges that can be manipulated under the influence of an external electric field. These surface charges are pH-dependent. Adjacent to the EDL lies the diffusive layer that extends into the bulk aqueous medium. The diffusive layer region consists of dissociated water ions that are loosely attracted to the biomass surface.^159^

Existing dewatering processes using membranes often employ a mechanical force (e.g., pressure), inducing liquid water to permeate the membrane.^160^ However, this mechanical force is nonspecific and is imposed on all species in the feed mixture, ultimately resulting in movement of all species in the slurry. This nonspecific behavior results in accumulation of biomass particles on the membrane surface and consequently pore blocking, thereby reducing the dewatering efficiency.

As biomass particles showcase electrostatic interactions at the biomass–water interface, they can be manipulated by superimposing an external electric field.^161^ The superimposed electric field forces selective migration of the charged aqueous phase surrounding the biomass through the membrane, while the biomass particles themselves are retained by the membrane. Based on the phase that moves, this selective migration can be classified as electroosmosis or electrophoresis.

#### 3.1.1 Electroosmosis

The EDL at the biomass–water interface has a charge opposite to that of the biomass.^116,117,158^ When an electric field is applied, the net charge in the EDL is forced to move, inducing a Coulombic force. As a result, the ions slip to the oppositely charged electrode, which is referred to as the slip plane. Subsequently, water in the bulk liquid adjacent to this slip plane moves along with these ions in the slip plane, resulting in water transport toward the oppositely charged electrode. This resulting flow is termed electroosmotic flow.

In the case of semiconcentrated biomass streams the effective electroosmosis extends beyond the membrane–aqueous interphase. It also becomes prevalent at the biomass–aqueous interphase. The exponential increase in effective surface area consequentially leads to an increase in the dewatering flow rate. This is sufficient to dewater the biomass stream to a significant concentration of 60–70%, as shown in the literature.^14,49^

**Figure 4:**
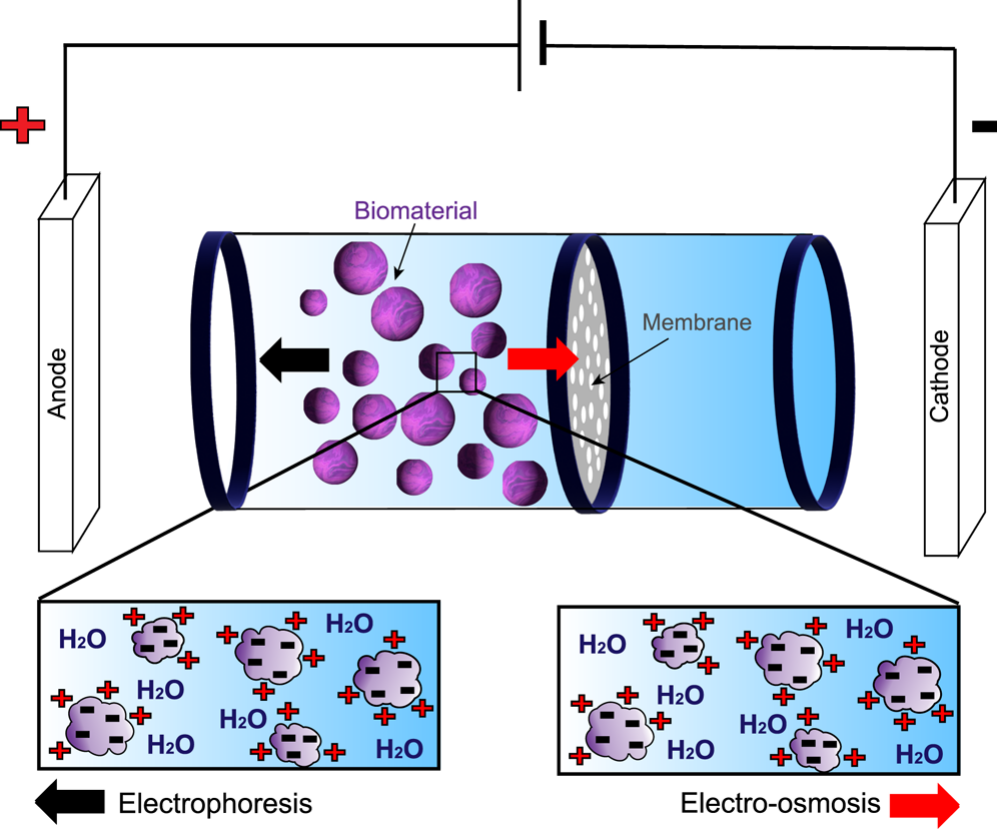
Selective migration of aqueous phase (electroosmosis) and biomass particles (electrophoresis) under the influence of a superimposed electric field.

The suspended particles thus remain stationary while the bulk liquid migrates.^162^ This migration of the diffusive layer and its adjacent bulk liquid is referred to as electroosmosis (EO) (Figure 4). EO is more powerful at higher biomass concentrations and especially when the charged biomass particles are porous and large enough to remain relatively stationary while the aqueous medium slips. This means that dewatering of biomass using an electric field is more effective at higher concentrations of biomass particles, producing a higher-quality dried product without degrading the biomass by shear or pressure.^14,49,61,163−165^

#### 3.1.2 Electrophoresis

Contrary to EO, electrophoresis (EP) is the movement of charged particles under the influence of an electric field (Figure 4). The electrophoretic mobility of these charged particles is a function of their charge density, size, and matter.^166,167^ Electrophoresis is more dominant in dilute slurries or with smaller biomass particles as, for similar surface charges, smaller biomass particles have higher charge densities than larger particles and are thus more affected by the presence of the electric field.^168^ Especially for biomass with small particle sizes, electrophoresis is an excellent technique for dewatering.^166,169^

Electrically assisted removal of water from biomaterial streams has the additional advantage over pressure driven processes that it is less susceptible to fouling. This is inherent to the chosen configuration; by choosing a certain electrode configuration and by tuning the direction of its electric field, we can migrate the charged biomass particles away from the membrane surface.^57,170,171^

#### 3.1.3 State of the Art

The literature presents several examples of the use of an electric field for the dewatering of biomass streams relying on EO or EP. Moreover, some studies have investigated the fouling sensitivity of membranes in electrically driven membrane processes. Both cases will be discussed below.

##### 3.1.3.1 Electric Field as a Driving Force

Holder et al.^166^ conducted experiments to separate peptides from a micellar casein hydrolysate using an electrically driven separation process. These peptides are distinctive in functional groups, peptide length, or charge and therefore show different electrophoretic movements under the influence of an electric field. The hydrolysate was passed through a UF membrane (PES, 5 kDa molecular weight cutoff (MWCO), and negative zeta potential) in a cross-flow configuration. The anionic peptides permeated through the membrane despite their negative zeta potential and migrated toward the positively charged anode, whereas the cationic peptides were retained in the feed as they were attracted toward the oppositely located cathode. The research also elaborated on the Coulombic forces experienced by differently sized peptides. Larger cationic peptides were retained at the cathode, while the smaller sized cationic peptides were pulled toward the membrane along with the convective flow of the permeate. The difference in behavior of the smaller and larger sized particles shows that this membrane separation method can be exploited for the fractionation of peptides. Similar findings were reported by Suwal et al.,^167^ who combined a PES UF membrane with an electrical potential in an electrodialysis filtration membrane process to fractionate antioxidant peptides using electrodialysis.

A study conducted by Chuang et al.^169^ reports the role of pH on surface charges during an electrically assisted membrane process. The effect of surface charges was investigated for an MF membrane (nylon-6,6) as well as a biomass mixture (yeast and BSA) in contact with an aqueous medium. The surface charge was varied by attachment of different functional groups to tune the surface charges, including opposite charges under the same pH conditions depending on the isoelectric point. The magnitude of these surface charges was measured through the zeta potential. Experiments conducted at pH 7 yielded a negative charge on both the membrane and biomass surface, whereas at pH 5 the two biomass components were oppositely charged (yeast negative and BSA positive), allowing for their charge separation. Acidifying the slurry further to pH 4 rendered the membrane surface positive, while the yeast component remained negatively charged. At pH 5, the oppositely charged membrane and BSA thus induce opposing electroosmotic flows, herewith reducing the overall permeate flux.

Concentration of fruit juice pectin is another application that can benefit from electrically driven dewatering. Sarkar et al.^172^ report enhanced concentration of fruit juice pectin by superimposing an electric field over a UF (50 kDa, PES) membrane. With the anode and cathode at the retentate and permeate sides, respectively, negatively charged pectin biomolecules were attracted to the anode and remained at the retentate side of the membrane. At the same time, the aqueous medium permeated through the membrane toward the cathode.

Where Sarkar et al.^172^ used a titanium anode and a stainless steel cathode, Munshi et al.^173^ conducted a similar experiment but employed stainless steel instead of titanium anodes. With the use of a noninert anode, ferric and ferrous ions were released to the aqueous medium. These ions were reported to bind with the microalgae in the feed, hereby reducing their surface charges and promoting their aggregation. Upon microalgae aggregation, the pore size of the bulk biomass reduced, improving the effectiveness of the electroosmotic dewatering process.

A study conducted by Poulin et al.^174^ compared a single cell and a four-cell-stack UF process in an electrodialysis setup to concentrate cationic peptides. The single cell was comprised of a UF (CE, 20 kDa molecular weight cutoff) membrane placed between an anion exchange membrane (AEM) and a cation exchange membrane (CEM). The four-cell stack was comprised of the same UF membrane sandwiched between two CEMs, repeated thrice, with the fourth stack ending near the anode. The permeate side of each cell was filled with a KCl solution, and the electrode chambers were filled with NaCl solution. The two configurations were tested with varying electric field strengths (2.75, 5.5, 11 V/cm). The results showed that a 4-fold increase in membrane area (four-cell stack) yielded a 4-fold increase in peptide concentration, irrespective of the applied voltage. The increase of the membrane area, by stacking four UF membranes, resulted in a 4-fold increase of the peptide concentration independent of the voltage value. However, for the four-cell stack, the concentration efficiency of peptides was reported to increase for the first 100 min and then reduce for the remaining operating time. This reduction in efficiency for the four-cell stack was explained by proton permeation through the CEM, thereby reducing the proton concentration in the feed chambers and thus increasing its pH. This increase in pH surpassed the isoelectric point of the peptides, which converted them into anionic biomolecules. Due to their negative charge, these biomolecules were no longer attracted to the cathode and thus did not permeate through the CEMs. This shows the importance of pH control in concentration and dewatering applications.

Cao et al.^164^ focused on concentrated slurries and investigated the dewatering of a concentrated algal slurry in an electrically assisted membrane process using PVDF membranes (pore size 0.45 μm). The study was conducted with different operational conditions, such as electric field strength and transmembrane pressure (TMP). A linear increase in dewatering efficiency with increasing electric field strength was observed, while with increasing TMP the dewatering first increased and afterward decreased again. This effect of the TMP was explained by eventual pore blockage induced by higher pressures that reduced the dewatering efficiency. The authors also found that an increase in ionic strength of the slurry resulted in screening of the biomass surface charges resulting in a reduced electroosmotic effect.

There are also studies that utilize electroosmotic dewatering of biomass combined with rollers to apply pressure on the already concentrated biomass to remove residual water trapped in the biomass pores. Raveendran Nair et al.^165^ reports a double function of these rollers as both pressure actuators and electrodes to obtain an improved dewatering of presoaked flax stems. In order to allow the double function, these rollers were made from porous carbon (pore diameter of 6 mm). The rollers, acting as an anode and a cathode, respectively, were placed above and below a cotton-made conveyor membrane. The presoaked flax stems were transported through the conveyor belt surrounded by closely placed cylinders applying a pressure of 10–30 bar and a potential of 12–36 V. The obtained results proved that pressure and voltage can be used interchangeably to increase the dewatering rate, whereas increasing the presoaking time had no effect after the flax stems were soaked for the first 12 h. Nair et al.^175^ performed a comparable study with hemp stems and obtained similar results.

Table 1 summarizes the above-mentioned literature sources that report enhanced separation efficiency due to selective migration of phases under the influence of an external electric field.

**Table 1:** Electrically Assisted Dewatering and Concentration of Biomass Slurry

biomass	membrane properties	electrically assisted process	separation performance	ref
ACE-inhibitory peptides	PES, UF 5 kDa, negative zeta potential	electrophoresis with anode on the permeate side	anionic peptides were sent to the permeate and small cationic peptides were moved to permeate due to convective flow	(166)
antioxidant peptides	PES, UF 20 kDa, surrounded by AEM and CEM	electrophoresis (0–60 V DC)	unwanted peptides permeate through the membrane due to convective flow of water	(167)
yeast and BSA	nylon-6,6, 0.2, 0.45 μm	electrophoretic separation	materials with different isoelectric points can lead to different charges and complicated electrophoretic migration	(169)
fruit juices	PES, UF 50 kDa, supported on PS-35	electrophoresis (200–800 V/m)	placing anode on permeate side increased flux by keeping large molecules away from membrane	(172)
bioactive peptides	CE, UF 20 kDa	electrodialytic fractionation (2.75, 5.5, 11 V/cm)	four-stack UF had faster migration rates than a one cell UF; better pH control required for consistent flux	(174)
algae	PVDF, 0.45 μm	electroosmotic dewatering	high ionic strength reduced algae zeta potential and high TMP compressed the algae closing pores for dewatering	(164)
flax stems	cotton cloth	electroosmotic dewatering using carbon rollers	TMP and electric field strength had similar effects on dewatering	(165)
hemp	cotton cloth	electroosmotic dewatering using carbon rollers	TMP and electric field strength had similar effects on dewatering	(175)

##### 3.1.3.2 Electric Field Enabled Antifouling

Kim et al.^57^ studied the fouling behavior of composite membrane electrodes used for electrically driven dewatering. Composite membrane electrodes were prepared by coating a selective layer of PVDF on a conductive carbon cloth. Under performance tests, the composite membrane electrode showed remarkable antifouling behavior toward microalgae particles. This could be attributed to the Coulombic repulsion between the negatively charged microalgae particles and the composite membrane electrode connected to the cathodic terminal of the DC power supply. Antifouling was further promoted by the hydrogen gas evolution reaction (HER) at the composite membrane surface, preventing biomass adsorption on its surface. In the case of biomass dewatering, the presence of solid particles reduces the feed ionic conductivity.^176^ This limits the extent of electrolysis achieved by applying an electric field. Therefore, we can consider electrolysis as a secondary process that assists in antifouling, without limiting the use of electric field as a driving force.

Similar antifouling behavior of a composite membrane electrode was observed by Huang et al.^171^ Also here, the composite membrane electrode was prepared by coating a selective layer of PVDF (produced using non-solvent-induced phase separation (NIPS)) on a stainless steel (SS) mesh electrode. Performance tests conducted for a membrane bioreactor slurry containing polysaccharides and proteins revealed high antifouling behavior of the synthesized composite membrane. The primary mechanism reported for this behavior was Coulombic repulsion between the membrane surface and biomass particles. This behavior was also supported by the formation of hydroxyl radical intermediates during the electrochemical reactions. The high oxidizing power of these radicals removed foulants from the membrane surface. Since these radicals have a short lifetime and are produced at the electrode surface, this mechanism works best with membranes that are either extremely close or fused to the electrodes.

Munshi^173^ investigated the effect of an electrical field in the feed chamber on water flux, fouling control, and algal morphology in the watering of algae streams using forward osmosis. Munshi used a cross-flow configuration with the algae feed at one side of the membrane and at the other side of the membrane a concentrated salt solution as a draw agent to induce a driving force for water permeation from the algae solution into the salt solution. An aquaporin thin film composite membrane was used. The results showed that the addition of an electric field induced Coulombic repulsion, which decreased fouling.

While the above examples elaborate on the antifouling performances of composite membrane electrodes, Dudchenko et al.^177^ reported the effect of membrane chemistry (i.e., carbon nanotubes) and physical properties (e.g., hydrophilicity) on the separation performance. Ultrafiltration composite membranes were synthesized by coating a 3:1 CNT-COOH:PVA layer on a polysulfone ultrafiltration support followed by cross-linking. Where adding PVA made the membrane hydrophilic and enhanced its antifouling behavior, CNT-COOH made the membrane conductive. The coated membranes showed lower fouling than the native polysulfone UF. Without an electric potential, fouling reduction was attributed to the more hydrophilic nature of PVA, making the coated membrane more hydrophilic. In the presence of an electric potential, Coulombic repulsion dominated the antifouling behavior of the coated membranes. However, too strong swelling of PVA can disrupt the conductive percolating network of CNT-COOH, hereby reducing the effect of Coulombic repulsion.

Considering Coulombic repulsions are a primary antifouling mechanism for electrically assisted membrane processes, several authors tried to mathematically describe these. Chuang et al.^170^ modeled this mechanism to evaluate the critical electric field strength required to remove any foulants from a membrane surface. The model was developed by balancing different force components acting on the foulant particle. Performance tests were used to separate poly(methyl methacrylate) (PMMA) from its colloidal suspension and revealed that the experimental critical electric field strength (60 V/cm) was higher than the modeled value (48 V/cm). This deviation between experimental and modeled values was attributed to the counter convective flow of water which also dragged the particles toward the membrane surface. These findings were used to improve the developed mathematical model, reducing the need for experimental evaluation of the antifouling performance.

Table 2 summarizes the above-mentioned literature sources that report enhanced antifouling due to the influence of an external electric field.

**Table 2:** Antifouling Performances for Electric Field Assisted Processes

foulant material	membrane properties	electrically assisted process	antifouling performance	ref.
microalgae *Chlorella*	composite membranes: PVDF coated, over a conductive carbon cloth electrode	electroosmotic separation	Coulombic repulsion, HER enabled antifouling	(57)
microbes, proteins, and polysaccharides	composite membranes: PVDF (0.06 μm) coated over SS electrode (96 μm)	membrane bioreactor	Coulombic repulsion and oxidation via hydroxyl radicals prevents fouling	(171)
algae	FO membrane: TFC aquaporin Sterlitech, coated over cathodic support	cross-flow forward osmosis	foulants were retained at the anode; shear flow was less effective compared to EF	(173)
PEO and alginic acid	composite membrane: PVA with CNT-COOH supported on PS-35	cross-flow ultrafiltration	EP had no effect on neutral foulants; hydrophilic membranes prevented fouling	(177)
PMMA colloidal suspension	nylon-6,6 with a negative zeta potential	cross-flow microfiltration	theoretical critical electric field strength, lower than experimental value	(170)

#### 3.1.4 Techno-economic Analysis

The literature examples shared above provide an overview of the performance enhancements of electrically induced membrane dewatering processes. These enhancements emerge from enhanced selective migration of components through the semipermeable membrane or from decreased fouling behavior helping to maintain the permeate flux sufficiently high in time. These arguments, while scientifically strong, are not sufficient for large scale implementation of electrically driven membrane dewatering processes. Techno-economic considerations are ultimately essential to address the feasibility for industrial acceptance.

Kim et al.^57^ provided detailed insight into the economic feasibility of superimposing an electric field over a cross-flow membrane dewatering process that is used to dewater algae. The findings suggest that a pressure driven UF process (3 kWh/m^3^) consumes more energy compared to its electrically assisted membrane dewatering counterpart (1.96 kWh/m^3^). This reduction in energy consumption makes electric field superimposition highly desirable, especially also when considering the much higher concentration factor achieved (6.47) compared to a pressure driven process (1.32).

Aoude et al.^56^ elaborates further on the energy consumed to dewater microalgae using electric field assisted membrane processes. The study accounts for energy losses due to ohmic heating at the electrode, providing a more comprehensive techno-economic analysis of the process. When compared to thermal dewatering techniques such as solar drying that consumes 2.35 kWh/kg of water removed, electrically assisted membrane dewatering only consumes 0.11 kWh/kg of water removed at a similar reduction in moisture content (15%). This suggests that electric field assisted membrane dewatering is more effective in obtaining dried products.

These examples lead us to believe that superimposing an electric field over a conventional membrane system can lead to an increase in the overall throughput while consuming less energy. Since electrical energy can be harvested from solar and wind, this also makes the process relatively sustainable.

### 3.2 Emerging New Materials: Hydrogels

Beside choosing a suitable membrane material, membrane surface treatment is an excellent way to optimize the membrane performance for biomaterial dewatering,^19,58,178,179^ as already mentioned in section 2. Hydrogels constitute widely investigated hydrophilic coatings that enable tunable permeation of water, organic solutes, and ions. In this section we give an overview of the characteristic properties of hydrogel membranes (HMs) and hydrogel composite membranes (HCMs), hereby indicating how the membrane performance can be enhanced by the use of hydrogels. We also include their fabrication methods and highlight the recently published strategies for biomaterial dewatering using HMs/HCMs.

Hydrogels are three-dimensional cross-linked polymer networks that are known for their ability to uptake large amounts of water.^180−184^ This hydrophilic nature is advantageous for dewatering applications, as it increases the permeation of water and decreases fouling of the membrane surface. In addition, their porous structure allows for selective diffusion of hydrophilic solutes into the material,^185^ rendering hydrogels highly suitable for application in agriculture, pharmaceuticals, catalysis, separation technology, biotechnology, and wastewater treatment.^186,187^ In fact, they have already been successfully produced at an industrial scale for some application fields, evident by the commercialization of hydrogel-containing contact lenses, wound dressings, and disposable diapers.^188^ By tuning the chemical properties, hydrogels can also be made responsive to various stimuli, such as redox chemistry, temperature, pH, and electric field.^181,189−191^ This stimuli-responsive nature may be useful to regulate the permeate flux and selectivity on-demand,^185^ as well as enabling the reuse of hydrogel draw agents.^192^ Lastly, hydrogels exhibit relatively smooth surfaces, which is suggested to prevent fouling in membrane applications.^178,180^ As such, hydrogels are an interesting class of materials to explore for dewatering processes.

Hydrogel membranes and hydrogel composite membranes are different from commercially available organic membranes due to their enhanced water absorption and dynamic mechanical properties.^180^ Bulk hydrogels display swelling ratios between 60 and 1450 g/g,^181,182^ whereas HMs generally show less swelling in an aqueous feed with values reported between 1 and 400 g/g.^193−195^ This degree of swelling is significantly higher than that of conventional water-permeable membranes such as PES,^42^ PVDF, and PSf.^180,196^ Furthermore, the tensile stress of HMs is known to decrease with increasing water content.^197^

**Scheme 1:**
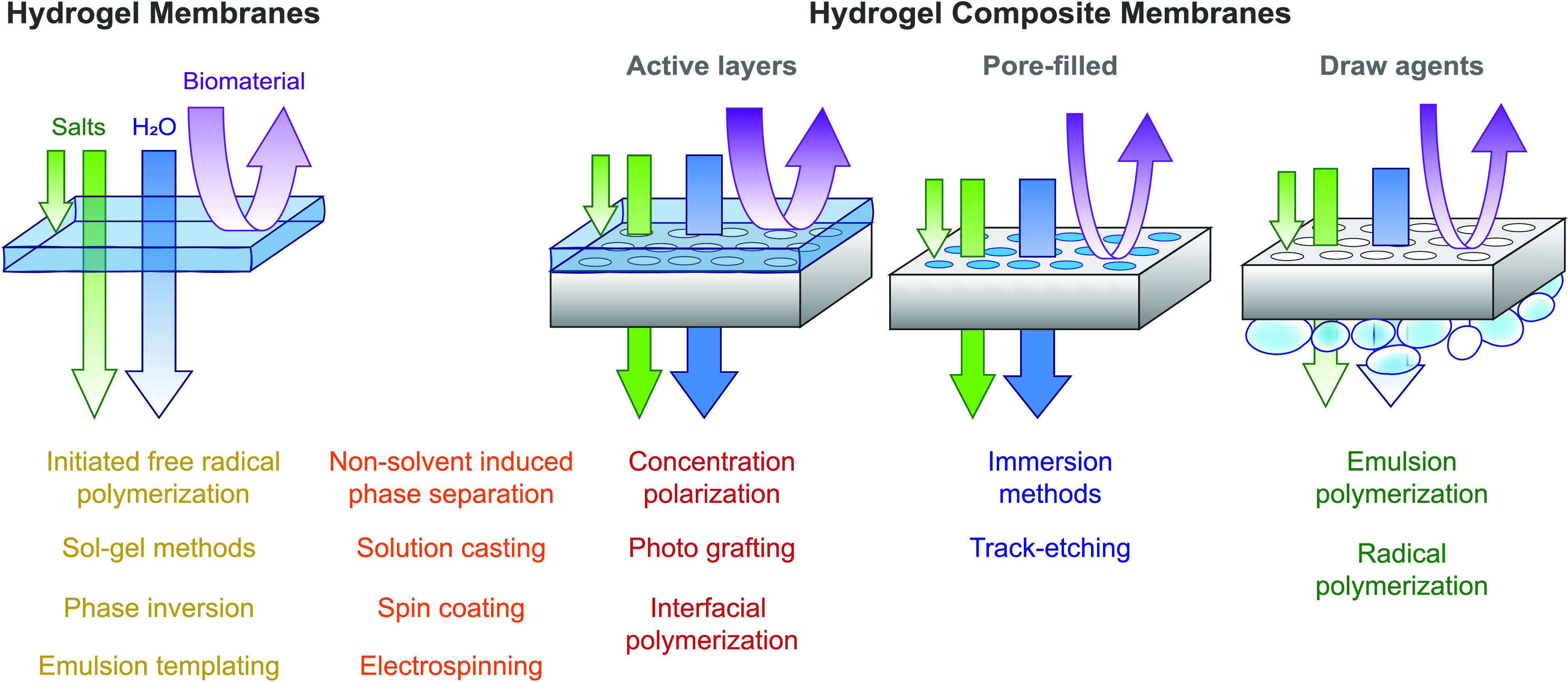
Schematic Overview of the Different HM/HCM Types and Their Fabrication Methods, Focusing on Dewatering Membranes Fabrication methods purely applied for HMs are indicated in yellow, while HCMs are divided into active layers (red), pore-filled membranes (blue), and draw agents (green). Overlapping methodologies for active layers and HMs are indicated in orange.

HMs and HCMs have been extensively used for a wide range of applications, such as tissue engineering, drug delivery, gas separation, ion exchange, and desalination.^26,179,180,198−202^ Upcoming application fields include wastewater purification^19,20,203,204^ and oil–water separation.^28,205,206^ Recently, various HM and HCM materials have been considered for dewatering applications. Herein, it is important to distinguish between HM and HCMs. HMs have a freestanding configuration^199,207−209^ or make use of a nonfunctional, porous support (Scheme 1).^41,185,210,211^ On the other hand, HCMs heavily rely on the separation properties of the supporting membrane.

In the literature, three major types of HCMs can be distinguished (Scheme 1). In the most common geometry a hydrogel coating on a functioning membrane acts as an active layer.^24,25,179,199,201,212−231^ In this geometry, the hydrogel functions as a selective separation layer^212,213,217^ or as an antifouling coating.^179,219,222^ Pore-filled membranes^232−238^ are porous membranes filled with a hydrogel material. The hydrogel typically acts as a component that enables tuning the separation efficiency via external stimuli such as pH^234,236,238^ and temperature.^236,239^

Hydrogels can also be synthesized in a bulk fashion to act as a soft draw agent.^23,52,184,192,200,240−244^ A draw agent is situated on the permeate side of a membrane and is able to attract water due to a difference in chemical potential. This creates a high osmotic pressure that mobilizes the water through the membrane.^245^ A major benefit of using hydrogels as draw agents is the ability to change the chemical potential via an external stimulus, such as temperature,^192,240^ and therewith enables reusing the material upon dewatering.^52^

#### 3.2.1 HM/HCM Properties

The internal structure of a hydrogel determines its suitability for diffusion-related applications^183^ and is strongly dependent on both the chemical composition and reaction conditions during synthesis. The separation efficiency and selectivity of hydrogels depend on the degree of cross-linking,^246−248^ a property that is determined by the relative amount of cross-linker compared to the polymer backbone. Typically, a higher cross-link density results in smaller pore diameters. The internal structure of hydrogels is typically found to be microporous, with pore sizes between 10 nm and 10 μm.^249,250^

Macroporous hydrogels are synthesized at low temperatures, by means of lyophilization or cryogelation.^251,252^ In such hydrogels, the internal structure is extremely porous, with pores larger than 10 μm in size.^249,250^ With increasing hydrogel pore size, the permeate flux and ion diffusion speed increase therewith allowing a faster response to stimuli.^249,251^ However, macroporous hydrogels exhibit a much lower mechanical stability and strength, requiring other strategies to ensure longevity.^249^ For dewatering applications, the design of supported HMs and HCMs has been investigated.^238^

The mechanical properties of hydrogels are strongly dependent on the chemistry, water content, and porous structure of the material^181^ and include characteristics like tensile strength, percent elongation to break, toughness, and Young’s modulus.^197,253^ The hydrogel mechanical properties can be enhanced by increasing the cross-link density,^181,253^ by incorporating second hydrogel networks,^181,254^ or by adding molecular stents^255^ or inorganic additives^256,257^ to the material. HMs often lack sufficient toughness, and thus they are often coated onto a porous support.^180,181^ Pore-filled membranes are also strongly dependent on the mechanical stability of the host membrane for convective flow applications.^246,247^ Herein, premodifying the pores with anchoring polymers has shown to improve the mechanical stability of the hydrogel.^239,258^

The permeate flux of HMs and HCMs is related to the pore size, pore size distribution, and hydrogel composition.^180^ The thickness of the hydrogel layer in HMs and active layers also plays a role, as thinner hydrogel films show higher permeate fluxes.^210^ For pore-filled membranes specifically, the permeate flux is highly dependent on the volume fraction of the hydrogel in the pores.^246,259^ Asymmetric membranes can be used to facilitate unidirectional diffusion of ions.^260^

HMs and hydrogel active layers bring another set of advantages which mainly involve antifouling. Hydrophobic attractions are responsible for deposition of protein biomass. Therefore, having a hydrophilic surface with polar moieties can render the surface inert, effectively stopping any fouling attractions.^261^ The inherent hydrophilicity and smoothness of hydrogels favor antifouling behavior.^97,101,262,263^ In addition, incorporating anionic or zwitterionic monomers in the material not only enhances the permeability for certain ions, it also enhances the antifouling properties for brackish water and biobased materials.^264−269^ Hydrogel coatings greatly reduce the surface roughness of membranes, which decreases the interfacial area for interactions between foulants and the membrane.^229,270^ Research on ultrasmooth hydrogel layers revealed that the smoother the hydrogel layer, the better the antifouling properties.^210^

#### 3.2.2 HM/HCM Fabrication

Hydrogel synthesis requires a monomer, an initiator, a cross-linker, and solvent.^181,183^ Copolymer, interpenetrating network (IPN), and double network (DN) hydrogels contain multiple monomers, which often leads to an enhancement of the water uptake^271^ and mechanical properties^254^ or induces certain stimuli-responsive properties.^208^

The water uptake ability of hydrogels depends on the hydrophilicity of the polymer network, which is induced by chemical moieties such as carboxylic (−COOH), hydroxylic (−OH), amidic (−CONH), and sulfonic (−SO_3_) groups.^184,186,253^ Smart HMs and HCMs are obtained by incorporating stimuli-responsive monomers.^185,272,273^*N*-Isopropylacrylamide (NIPAM) is a widely used thermoresponsive monomer,^24,189,192,200,252,274−277^ while *N*-vinylisobutyramide (NVIBA) provides a suitable alternative for biomedical applications.^278^ Methacrylic acid (MAA),^238^ acrylic acid (AA),^207,234,279^ and *N*,*N*-dimethylaminoethyl methacrylate (DMAEMA)^226,265^ are commonly used pH-responsive monomers. HM and HCM materials have been reported to dynamically and reversibly change their permeability or antifouling properties with temperature,^24,224,274,276,280^ pH,^207,228^ pressure,^280^ electric field,^215^ and light.^281,282^

The introduction of covalent bonds between polymer chains using small organic molecules such as glutaraldehyde is the most commonly used cross-linking technique in HM and HCM fabrication.^20,181,186^ Radical polymerization and cross-linking is the second most used method.^20^ Both techniques yield chemically cross-linked hydrogel materials, with enhanced mechanical strength, thermal stability, swelling properties, and durability as compared to physically cross-linked alternatives.^20,253,283^ Small cross-linker molecules are primarily mono- and bifunctional,^181^ with an exception being tannic acid as a multifunctional cross-linker.^23,223,284^ Radical polymerization reactions often make use of bifunctional vinyl cross-linkers.^185,195,285^

Reportedly, some HCMs have been fabricated with additional inorganic or hybrid fillers. Additives can be incorporated into hydrogel matrixes to enhance their mechanical, dielectric, and antifouling properties or thermal stability.^286−288^ For example, Ali et al.^287^ showed that the addition of Al_2_O_3_ and SiO_2_ fillers to PVA/PVP HCMs enhances the dielectric properties. On the other hand, calcium phosphorus (CaP) fillers improved the mechanical properties of gelatin membranes.^288^ Silver–polydopamine (Ag–PDA) nanospheres were coated onto a PSF support to enhance the water flux and antibacterial properties while maintaining a good antifouling membrane surface.^289^ In some recent publications, graphene oxide (GO) nanosheets were blended into active layers of P(VSA-*co*-METMAC)^230^ and PVA-SA^290^ to promote antifouling and antibacterial activity.

The synthesis of hydrogels is typically done in aqueous liquids.^41,180,207,208,219,291,292^ On the other hand, changing the solvent to a binary mixture can enhance the hydrogel material properties. For instance, it was demonstrated by Sadeghi et al.^211^ that including PEG additives in the aqueous solution enhances the porosity of hydrogel membranes. Xu et al.^293^ used mixtures of water and ethylene glycol (EG) to induce partial polymer phase separation during polymerization and subsequently create opaque, loofahlike hydrogels. Zhao et al.^252^ used PVA dispersants to control the pore size of macroporous hydrogels.

The choice of fabrication methods for HMs is limited, as the goal is to either yield freestanding membranes or to ensure adhesion of the hydrogel thin film to an underlying support (see Scheme 1).^180^ Common HM fabrication approaches in the literature include spin coating,^198,294^ emulsion templating,^295^ phase inversion,^41^ sol–gel methods,^281^ and solution casting.^27,29,185^ Herein, solution casting is the most commonly used method due to its facile nature. UV irradiation is a frequently used follow-up step to induce gelation of the hydrogel.^185,198^

Electrospinning is an upcoming method for the fabrication of HMs and is used to obtain more flexible membranes,^296^ with enhanced hydrophilicity,^218^ biocompatibility,^297^ and fast pH-responsive membranes.^207^ Other upcoming techniques for HM fabrication include interfacially initiated free radical polymerization (IIFRP),^211,213^ NIPS,^23^ and macroinitiator-mediated photopolymerization.^219,221^

For each of the HCM geometries, a certain set of fabrication methods has been used (see Scheme 1). Active layers were typically fabricated through casting,^199,212,214,226,298^ electrospinning,^218^ spin coating,^220^ concentration–polarization,^221^ interfacial polymerization (IP),^201,225,227,229^ photografting,^222^ or NIPS.^217,230,231^ On the other hand, pore-filled membranes have been prepared by track etching,^224,235,239^ or by immersion in precursor solution, followed by polymerization through UV irradiation^232,233,237,299^ or heat.^234^ Lastly, draw solutes can be made by hydrogel bulk synthesis procedures, such as radical polymerization^192,241,242,244^ or emulsion polymerization.^200^ Herein, stirring is often maintained throughout the gelation process to ensure the formation of homogeneous hydrogel particles.^192^ Alternatively, postprocessing was done by cutting^241,242^ or grinding the hydrogels after drying.^52,244^

For supported HMs, active layers, and pore-filled HCMs, anchoring of the hydrogel to a surface is crucial. Weak attachment typically leads to delamination failure due to the dynamic swelling of hydrogels. Conventionally, noncovalent interactions dominate the adhesion of hydrogels to a surface. Examples include physical and ionic attractive forces.^219^ Explicit adhesion of hydrogels has been achieved both noncovalently and covalently, by physical absorption of initiator molecules and by plasma treatment of the supporting membrane, respectively.^219,300^ A few recent studies have focused on the prevention of delamination using charged macroinitiators for photografting,^219,221,301^ Schiff base chemistry,^302^ catechol chemistry for a covalent grafting-from approach,^303^ and click chemistry for a covalent grafting-to approach.^304^

#### 3.2.3 HM/HCMs for Dewatering

In Table 3, an overview is provided of HM and HCM materials used for dewatering. The research on HM and HCM materials can be divided into three different categories: I, fundamental research of HM/HCM materials; II, research into the antifouling properties of HM/HCM materials; and III, HM/HCM systems for dewatering. These categories are included per study in Table 3 as a guide to the reader.

**Table 3:** HM/HCM materials, Configuration, Feed, and Performance, Sorted on Different Types of Research Categories ( I–III)^a^

cat.	material	config	feed	performance	ref
I	PVA	S-PES/GO	glycerol, glucose, sucrose, raffinose, Na_2_SO_4_ (0.5 M)	ultrathin membranes increase water flux 10 times; ultrasmooth membranes enhance antifouling	(210)
I	P(AA-*co*-HEMA)	S-PP	HA, BSA (10 mg/L)	NaCl decreases the hydrogel thickness, which lowers mass transfer resistance	(185)
II	PVA	S-PES/GO	HA, SA, *n*-hexadecane, BSA (2–20 g/L)	separation efficiency is 2 times higher than commercial desalination membranes	(41)
II	PEGDA	S-PS	BSA, cytochrome *c* (100 ppm)	PEGDA is found suitable for protein purification, with a <2% flux reduction in protein feed demonstrate lowest adsorption of proteins	(211)
II	PSBMA	AL-PA	HRP-conjugated goat antihuman IgG (3 mg/mL)	antibody absorption reduced by 97%, water flux increased by 65%	(229)
II	PEGMEMA	AL-PA	BSA, Lys (10 g/L)	macroinitiated membrane fabrication yields good antifouling properties and prevents delamination	(219)
II	sodium alginate	AL-PA	BSA (1000 ppm), DTAB (50 ppm) *S. aureus*, *E. coli* (OD_600_ 0.3)	water flux increased by 192.97%; enhanced antifouling by 26–30%; shows antibacterial properties	(225)
II	P(SBMA-*co*-MAHEMA)	AL-PA	BSA, Myo (1 g/L)	hydrogel layer causes a decrease in protein absorption and increase in water flux	(221)
II	P(VSA-*co*-METMAC) (additive: GO sheets)	AL-PES	BSA (1 g/L), SMP (10 ppm)	zwitterion polyampholyte-modified membranes show good antifouling and antibiofouling	(230)
II	PEGDA-SBMA, PEGDA-PEGA, PEGDA-MPC	AL-PS	soybean oil (1.5 g/L), Myo (0.1 g/L)	incorporating zwitterions in the selective layer increases permeance; no effect on Myo rejection	(269)
II	PEGDA-SBMA	PF-PTFE (PA skin)	Na_2_SO_4_, BSA (1 g/L)	no ICP when PEGDA-SBMA is introduced; PA skin improves water/salt selectivity	(237)
II	P(NIPAM-*co*-DEM)	DA	BSA (100 mg/L), SA (20 mg/L), octanoic acid, (20 mg/L) NaCl (17 mM), CaCl_2_ (1 mM)	constant water flux of 1.90 LMH achieved for comonomer DA	(192)
III	PAA-*co*-PNIPAM (additive: CMC)	DA-CTA	BSA (0.1 g/L)	regeneration possible by heat; recycled gels show 5% loss in flux	(240)
III	PNIPAM	DA	algal slurry	hydrogel acts as DA for water and responds to heat and CO_2_	(243)
III	cellulose	DA	orange juice	total of 31% dewatering is achieved	(52)

a The geometries are labeled as follows: S, supported HM; AL, active layer HCM; PF, pore-filled HCM; DA, draw agent HCM.

**Figure 5:**
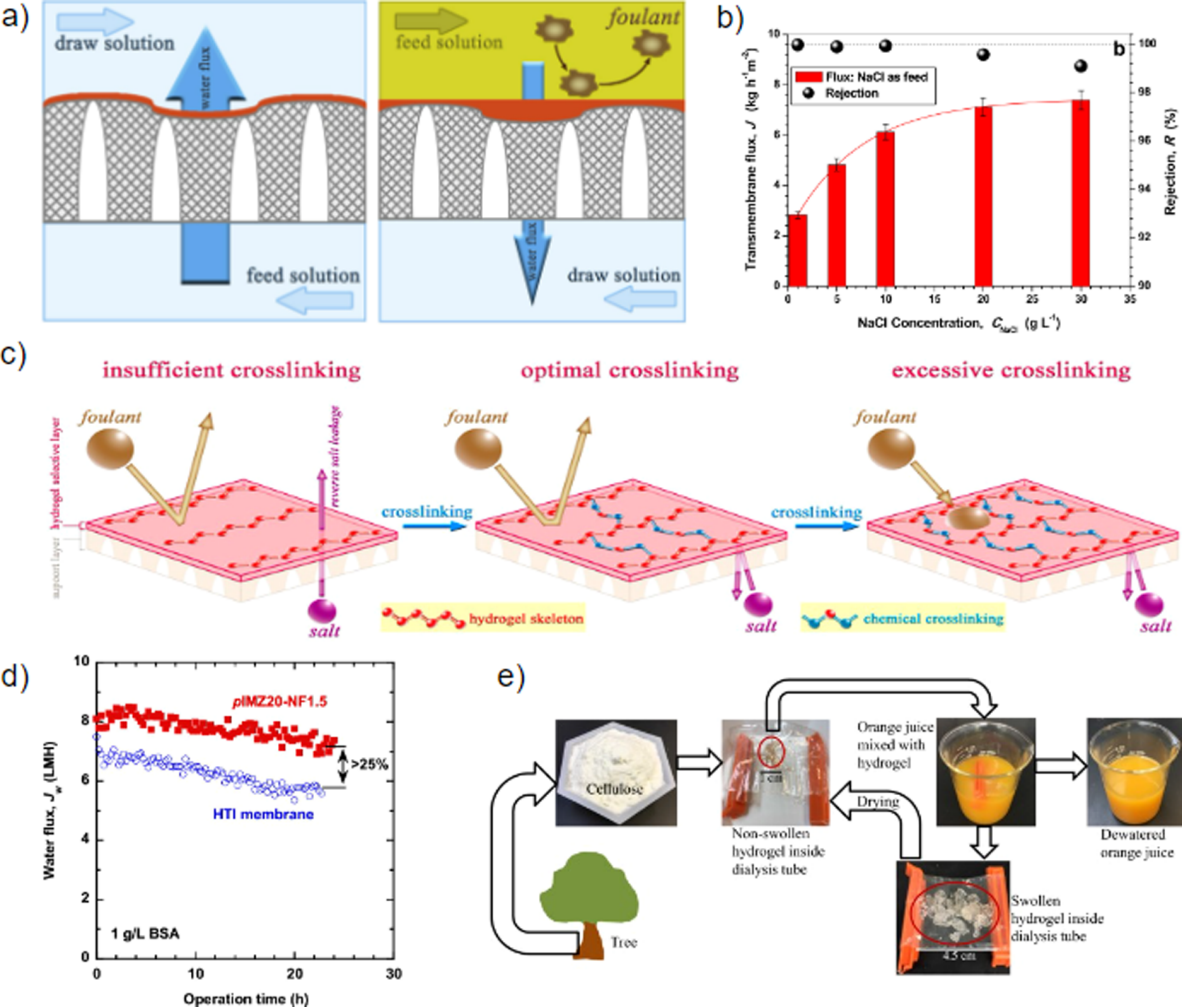
(a) Fine-tuning of the architecture yields ultrathin (left) and ultrasmooth (right) hydrogel selective layers. Ultrathin layers show enhancement of water flux, while ultrasmooth layers promote antifouling behavior. Adapted with permission from ref (210). Copyright 2019 Elsevier. (b) The transmembrane flux (left *y*-axis) and salt rejection of a P(HEMA-*co*-AA) membrane at varying NaCl concentrations. Reprinted with permission from ref (185). Copyright 2016 Elsevier. (c) The effect of cross-linking density on the antifouling capability and separation efficiency of hydrogel forward osmosis membranes. Reprinted from ref (41). Copyright 2018 American Chemical Society. (d) Water flux of commercial membranes (HTI) compared to pore-filled HCMs (pIMZ30-NF1.5), showing an increased water flux for the investigated HCMs of >25%. Reprinted from ref (237). Copyright 2020 American Chemical Society. (e) Dewatering mechanism of orange juice by means of cellulose hydrogels. Reprinted with permission from ref (52). Copyright 2020 Springer Nature.

Fundamental research on HM and HCM materials has focused on the role of hydrogel layer architecture and salt presence for both membrane transport properties and antifouling. Qin et al.^210^ prepared PVA membranes with varying thicknesses and surface roughness on a PES/GO support and determined their permeability and permselectivity. It was revealed that 45-nm-thick hydrogel layers achieve the maximum separation efficiency and that ultrasmooth membranes (<1 nm variation) promote antifouling properties (Figure 5a). The relation between HM mass transport properties and the presence of salts was investigated by Majidi Salehi et al.,^185^ who prepared supported P(AA-*co*-HEMA) membranes to remove excess water from 10 mg/L humic acid (HA) and bovine serum albumin (BSA) solutions. It was found that the water flux increases for higher ion concentrations and inherently thinner HMs. Oppositely, the selectivity for water transport is increased for thicker membranes (Figure 5b).

The majority of the studies on HM/HCM membranes for dewatering have focused on the enhancement of antifouling properties by using various model foulants, including BSA, HA, sodium alginate (SA), dodecyl trimethylammonium bromide (DTAB), lysine (Lys), myoglobin (Myo), cytochrome *c*, and *n*-hexadecane. Enhancing the antifouling properties was typically achieved by the introduction of zwitterionic hydrogel layers.^221,229,230,237,269^ Other strategies involved the use of advanced membrane fabrication techniques such as IIFRP,^211,269^ layer-by-layer grafting,^225^ mediated photografting,^219^ or concentration polarization (CP).^221^

The research on antifouling HM/HCM materials for dewatering provides more insight into the role of hydrogel chemistry in the fouling behavior and membrane performance. For instance, Qin et al.^41^ found that there is an optimal cross-link density, at which a PVA membrane shows good antifouling behavior and no salt leakage (Figure 5c). Tran et al.^237^ prepared pore-filled poly(tetrafluoroethylene) (PTFE) membranes to circumvent the well-known issue of internal concentration polarization (ICP) in porous membranes (Figure 5d). Herein, zwitterionic hydrogels of poly(ethylene glycol) diacrylate (PEGDA) and SBMA were used as a filler and PA selective layers as a way to promote antifouling.

Finally, a select few papers have reported on the dewatering performance of HM/HCM systems. Gawande et al.^240^ synthesized a draw agent based on PAA–PNIPAM for the enrichment of BSA protein solutions. The recovery and reusability of the draw agents were tested via thermal dewatering and showed a 5% loss in flux. Vadlamani et al.^243^ patented the use of stimuli-responsive hydrogels for the harvesting of microalgae. The patent includes the specific use of semi-IPN hydrogels made from PNIPAM. Regeneration of the hydrogels is achieved via heat or CO_2_. Islam et al.^52^ were the first to explicitly describe a *dewatering* process with the use of hydrogel draw agents. Cellulose hydrogels were used to dewater orange juice, where a dewatering yield of 31% was achieved (Figure 5e). The dewatering process itself was conducted at small scale, whereby the draw agents were placed into dialysis tubes. Regeneration of the draw agents was done up to three times and was achieved by drying the gels at 50–60 °C.

## 4 Summary and Outlook

Our literature review reports on recent advances in membrane separations for the dewatering of biomaterials. First, we identified a set of requirements for efficient dewatering membranes tailored for biobased feeds and discussed how they can be controlled with different design parameters. Next, we focused on two upcoming advances in this field, which are (I) electrically driven membrane systems and (II) membrane surface functionalizations with hydrogels.

In summary, four design requirements are essential for efficient dewatering membranes, which are related to their permeate flux, selectivity, antifouling properties, and scalability. A high membrane performance is characterized by a combination of low fouling with a high selectivity and permeate flux. These performance parameters can be optimized by adapting the membrane chemistry and morphology. From a chemistry perspective, membranes should possess hydrophilic moieties and surface charges to enhance their dewatering performance and reduce fouling. Membrane morphology is equally important, wherein high porosity and sufficiently small pore sizes are needed for an optimal permeate flux and sufficient selectivity, while a low surface roughness is desired for minimal fouling. Lastly, the fabrication and operation of such membranes should be durable, scalable, and cost-effective to be able to compete with the conventional thermally driven processes.

Recent developments in the field of membrane dewatering have focused on two different strategies, one of which depicts the application of electric fields. Electrically assisted membrane dewatering induces selective migration of biomaterial components and antifouling behavior, herewith enhancing the dewatering efficiency.

Electrically driven dewatering studies have been conducted with well-defined peptide feeds and more complex biomaterials such as fruit juice, algae, and plants. This shows us that electrically assisted dewatering is an up-and-coming field that has moved beyond fundamental studies with model feeds and model foulants.

The majority of these studies report on electrically assisted dewatering of diluted biomaterial slurries, where electrophoresis is the driving force. Herein, researchers have investigated the role of feed conditions, such as pH and ionic strength, and their membrane configuration, such as membrane stacking and electrode positioning. In parallel, antifouling membranes were obtained with conductive membrane electrodes that repel foulants by Coulombic repulsion.

Our literature overview shows that electrically assisted dewatering is largely investigated for diluted biomaterials rather than concentrated slurries. Therefore, electroosmotic dewatering with membranes is not yet explored for an extensive range of slurry conditions and compositions. Challenges for the future also lie within this field and in the selection of suitable membranes for electroosmosis.

The use of hydrogel membranes is another advancement in the field of membranes, which is relatively unexplored for dewatering applications. A promising foundation has been provided by several studies that employ ions, model foulants, and protein solutions such as sodium chloride, BSA, and HA, with the primary goal to provide a better understanding of the structure–property relations of freestanding and supported hydrogel membranes. Structural changes were often induced by tuning the monomer composition or cross-link density, while performance tests were focused on fouling properties and water flux.

Hydrogel composite membranes have received considerably more attention. Their geometries can be divided into three categories: supported active layers, pore-filled membranes, and draw agents. Especially hydrogel draw agents have been investigated more in the context of dewatering, with the first studies with biomaterial feed being performed. From these recent studies, we conclude that active layers tend to improve antifouling properties and permeability and that pore-filled membranes are often employed in stimuli-responsive systems. Lastly, draw agents have already shown to be suitable for the dewatering of orange juice and algal slurry.

Research on hydrogel membranes and hydrogel composite membranes is currently in the fundamental stages, using model systems and lab scale equipment. To assess the suitability of hydrogel (composite) membranes for biomaterial dewatering, more research should focus on the performance of such membranes in more realistic model feeds that mimic biomass or represent biomass more accurately. In addition, the processes have not been scaled up to industrial scale yet, which requires more knowledge on mechanical strength and long-term durability. Such tests should also be performed in order to understand which design requirements are especially relevant for hydrogel (composite) membranes. Ideas and suggestions for such improvements can be taken from the field of oil/water separations or desalination, where hydrogel membranes are more extensively investigated and tested in large scale systems.

From the existing literature on dewatering membrane materials and recent advances, we have concluded that the field still faces major challenges with fouling and slurry dewatering efficiency foremost. Solutions to these challenges come in various ways, including techniques like electrically driven systems and HCM materials that have shown great promise so far. We believe the future of membrane dewatering also lies in these developments and the maturing of such techniques to be able to sustainably dewater biomaterials at industrial scale and herewith smoothen the transition to a biobased economy.
